# Naturally occurring diversity helps to reveal genes of adaptive importance in legumes

**DOI:** 10.3389/fpls.2015.00269

**Published:** 2015-04-21

**Authors:** Laurent Gentzbittel, Stig U. Andersen, Cécile Ben, Martina Rickauer, Jens Stougaard, Nevin D. Young

**Affiliations:** ^1^EcoLab Laboratoire Écologie Fonctionnelle et Environnement, Institut National Polytechnique de Toulouse, Ecole Nationale Supérieure Agronomique de Toulouse, Université Fédérale de ToulouseCastanet Tolosan, France; ^2^EcoLab Laboratoire Écologie Fonctionnelle et Environnement, Centre National de la Recherche ScientifiqueCastanet Tolosan, France; ^3^Department of Molecular Biology and Genetics, Centre for Carbohydrate Recognition and Signalling, Aarhus UniversityAarhus, Denmark; ^4^Department of Plant Pathology, University of MinnesotaSt. Paul, MN, USA; ^5^Department of Plant Biology, University of MinnesotaSt. Paul, MN, USA

**Keywords:** *Medicago truncatula*, *Lotus japonicus*, *Glycine max*, GWAS, genetics, whole genome sequencing, gene expression

## Abstract

Environmental changes challenge plants and drive adaptation to new conditions, suggesting that natural biodiversity may be a source of adaptive alleles acting through phenotypic plasticity and/or micro-evolution. Crosses between accessions differing for a given trait have been the most common way to disentangle genetic and environmental components. Interestingly, such man-made crosses may combine alleles that never meet in nature. Another way to discover adaptive alleles, inspired by evolution, is to survey large ecotype collections and to use association genetics to identify loci of interest. Both of these two genetic approaches are based on the use of biodiversity and may eventually help us in identifying the genes that plants use to respond to challenges such as short-term stresses or those due to global climate change. In legumes, two wild species, *Medicago truncatula* and *Lotus japonicus*, plus the cultivated soybean (*Glycine max*) have been adopted as models for genomic studies. In this review, we will discuss the resources, limitations and future plans for a systematic use of biodiversity resources in model legumes to pinpoint genes of adaptive importance in legumes, and their application in breeding.

## Introduction

The legume family (*Leguminosae*) is second only to cereals in economic and nutritional value. Grain legumes, such as common bean (*Phaseolus vulgaris*), lentil (*Lens culinaris*), or chickpea (*Cicer arietinum*) provide on average 33% of human's dietary nitrogen and up to 60% in developing countries (O'Rourke et al., 2014). Altogether, grain legumes are grown on 115 million ha, with soybean for feed and food grown on 111 million ha, representing 276 million tons. The major fodder legume, alfalfa, is cultivated over 15 million ha for 340 million tons of forage^1^.

**Figure 1:**
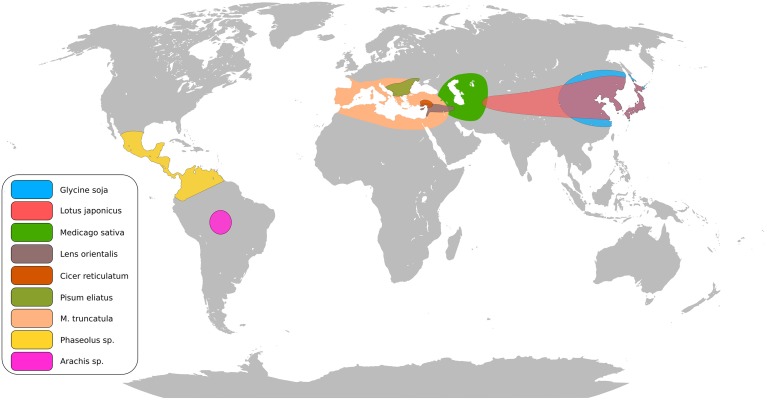
**Putative centers of origin of major legume crops and model legumes**.

The legume family consists of approximately 20,000 species (Doyle and Lucknow, 2003). Legumes are often pioneer plants improving soil fertility and moderating harsh conditions, as demonstrated with *Lotus corniculatus* (Esperschütz et al., 2011). Figure 1 summarizes the centers of origin for several important cultivated and model legumes. Legumes establish symbiotic interactions with both rhizobia (the *Rhizobium*-legume symbiosis, RL) and arbuscular mycorrhizal (AM) fungi leading to formation of nitrogen-fixing nodules and phosphate acquiring mycorrhiza (Oldroyd, 2013). These traits play a vital role in ecosystems and sustainable crop production, and are central for efforts to decrease dependence on commercial fertilizers and substitute imported protein feeds with locally produced legume crops (Voisin et al., 2014). Aiming to uncover the genetic basis for these features, *Medicago truncatula* and *Lotus japonicus*, have come to supplement *Arabidopsis*, maize and rice as plant models. Through a mutant approach a core set of RL symbiosis genes has been identified in the two model legumes (Kouchi et al., 2010; Gough and Cullimore, 2011). Virtually all current knowledge about the perception of rhizobial signals, transduction and organogenesis originates from these studies. The RL and AM signaling pathways overlap and the genetic basis of this common symbiosis pathway comprises more than 10 genes (Parniske, 2008). Several mutants affected in RL symbiosis also show altered responses to root pathogens (Rey et al., 2013; Ben et al., 2013b) suggesting crosstalk between signaling pathways in symbiosis and diseases.

Translational biology (from models to crops) is still in its infancy. This could be due to lack of genetic variability, where the crop's orthologous gene is not polymorphic and thus not amenable to breeding. Strategies to introduce heterologous genes into crops may encounter technical difficulties, or societal issues such as consumers' acceptance. Genes identified in mutant-based approaches may also be unrelated to adaptation and responses to biotic and abiotic stresses encountered in agriculture or natural environments. Initial phenotypic screens may not have been set up to target “adaptive” traits such as seed yield, biomass, or fitness. Nonetheless, legume crops do benefit from models: the RCT1 gene from *M. truncatula* provides resistance to anthracnose in alfalfa (Yang et al., 2008). Wild species also contributed to the improvement of crops: a high-protein allele has been introgressed into soybean [*Glycine max* (L.) Merr.] from its wild progenitor, *Glycine soja* Sieb. and Zucc (Sebolt et al., 2000; Vaughn et al., 2014).

Many legume crops have limited genetic diversity due to bottlenecks from domestication and selective breeding (Gross and Olsen, 2010; Roorkiwal et al., 2014). Proper characterization and evaluation of germplasm collections as sources of adaptive alleles, and their utilization in breeding are often limited or neglected (Smýkal et al., 2015). However, the anticipated climate change may require explicit efforts to breed for local adaptation. The use of wild relatives of cultivated legumes crops has received limited attention as well. As a consequence, selection of new cultivars and improving production technologies for food legume crops are not proceeding at the same pace as for cereal crops.

*M. truncatula* and *L. japonicus* as members of the *Hologalegina* clade (which contains cultivated legumes such as pea, chickpea), and *G. max* as a representative of the *Phaseoloid* clade (containing mungbean and soybean) can serve as models for cool season and tropical season legume crops, respectively (Doyle and Lucknow, 2003; Zhu et al., 2005). The high degree of synteny between these model plants and their legume crops relatives can been exploited to improve genetic maps and identify candidate genes for agronomic traits in less well-characterized crops, as has been described for leaf size in cowpea (Potorff et al., 2012), drought adaptation traits in faba bean (Kharzaei et al., 2014) or symbiotic genes in pea (Novak et al., 2012). In this Perspective, we will discuss the resources, limitations and future plans for a systematic use of biodiversity resources in model legumes to pinpoint genes of adaptive importance in legumes.

## Exploring the natural diversity of *Medicago truncatula*

*Medicago truncatula* is closely related to alfalfa. Together with *L. japonicus*, *M. truncatula* has been essential for studies of RL and AM symbioses. In addition, agronomic and quality traits (Julier et al., 2007), including drought and salinity tolerance (Friesen et al., 2014), seed development and composition (Vandecasteele et al., 2011), and disease resistance (e.g., Ben et al., 2013) have been targeted. Consequently, this species has an excellent array of germplasm, molecular, and genomic resources.

Studies of *Medicago* populations focused on collections from the Mediterranean basin, its center of diversity. Work in France led to the establishment of extensive germplasm resources (Thoquet et al., 2002), including accessions covering *M. truncatula*'s natural range plus strategically selected biparental crosses and derived recombinant inbred lines. Additional material is available from SARDI (Ellwood et al., 2006) and USDA-GRIN. Another valuable germplasm resource is the *Tnt1* insertion population created at the Noble Foundation, which provides knockout mutants for reverse genetics and characterization of candidate genes (Pislariu et al., 2012). The genome sequence released in 2011 (Young et al., 2011) and refined in 2014 (Tang et al., 2014) established *M. truncatula* as an outstanding system for genome studies. Altogether the Mt4.0 assembly covers 360 Mb and gene annotation uncovers 31,661 high confidence predicted genes.

The underlying genome architecture was first described in Branca et al. (2011) using 26 accessions broadly spanning *M. truncatula*'s range, at 30x sequence coverage. This uncovered four to six SNPs/kb and LD decays reaching half its initial value within 3–4 kb, similar to that of *Arabidopsis* (Kim et al., 2007).

More than 20,000 annotated genes from 56 accessions were used to identify targets of positive selection (Paape et al., 2013). Around 1% of sampled genes harbored a signature of positive selection, while 50–75% of non-synonymous polymorphisms were subject to purifying selection. Among putative targets of selection were genes involved in defense against pathogens and herbivores and in relationship with rhizobial symbionts.

Local adaptation and adaptive clines were examined in 202 accessions at 2 million SNPs, identifying loci responsible for adaptation to climatic gradients: annual mean temperature (AMT), precipitation in the wettest month (PWM), and isothermality (Yoder et al., 2014). The strongest associations tagged genome regions containing genes with predicted roles in tolerance to temperature, drought, herbivores or pathogens. The candidate loci were further tested and validated using climate-controlled tests. For AMT and PWM, a history of soft selective sweeps acting on loci underlying adaptation was indicated.

GWAS based on 6 million SNPs, identified by re-sequencing 226 *M. truncatula* accessions, revealed candidate genes underlying phenotypic variation in several plant functional traits (Stanton-Geddes et al., 2013). For flowering time and trichome density, peaks were associated with well-supported candidates (MtFD for flowering time; *unshaven* in the case of trichome density). For rhizobium symbiosis, previously characterized nodulation genes (SERK2, MtnodGRP3, MtMMPL1, NFP, CaML3, MtnodGRP3A) were confirmed, and novel loci were identified with annotation and/or expression profiles that supported a role in nodule formation.

Population genomics revealed candidate regions associated with local adaptation (Friesen et al., 2010, 2014) to saline environments, by searching for SNP that assorted by population i.e., two populations from saline environments vs. two from non-saline environments. The analyses pinpointed signaling pathways for abiotic stress tolerance involving ABA and MeJA, production of putative osmoprotectant, and candidates linked to biotic interactions. A strategy for salt stress avoidance through early flowering was suggested, specifically a non-synonymous SNP that changes a highly conserved amino acid in the *Medicago* ortholog of *CONSTANS* (Pierre et al., 2011).

*M. truncatula* is a host for leaf and soil-borne pathogens (Ameline-Torregrosa et al., 2007; Ramírez-Suero et al., 2010; Rispail and Rubiales, 2014). Quantitative resistance loci for root diseases have been described for the interaction with *Ralstonia solanacearum* (Ben et al., 2013a), the oomycete *Aphanomyces euteiches* (*Ae*) (Djébali et al., 2009; Pilet-Nayel et al., 2009), and *Verticillium alfalfae* (Ben et al., 2013b). Bonhomme et al. (2014) utilized GWAS to map loci associated with *Ae* resistance. This identified several candidate genes and pinpointed two major loci on chromosome 3, that co-located with previously described QTLs (Djébali et al., 2009; Pilet-Nayel et al., 2009). Intriguingly, the resistance allele of the candidate gene seemed to be the ancestral form, although the pathogen and the resistant host plant do not occur naturally in the same geographical regions. Another study of *Ae* resistance with 136 lines from 14 Tunisian populations, not naturally exposed to the oomycete, suggested that resistance can occur as a by-product of adaptation to water stress (Djébali et al., 2013).

Clearly, the evaluation of naturally occurring variations in *M. truncatula*, making use of its well-characterized biodiversity and feature-rich genomic tools, is becoming a powerful strategy of investigation. Identification of candidate genes for major traits is likely to expand rapidly.

## Lotus resources for functional analysis of natural diversity

Several *Lotus* species are cultivated as forage legumes, especially in grassland areas characterized by harsh environments, including windy and salty coastal climates (Escaray et al., 2012). The genetic background for such plasticity and adaptation to adverse conditions in poor soils, flooding, drought, salt and other abiotic stresses is important for plant production in a world facing climate changes. Model plants are essential for unequivocal identification of genetic regulators, and one of the diploid and self-fertile Lotus species, *L. japonicus*, was proposed in 1992 as a model system for classical and molecular genetics (Handberg and Stougaard, 1992). Since then, genetic and physical maps, F2 and recombinant inbred line populations, transformation protocols and a reference genome sequence have been established (Hayashi et al., 2001; Kawaguchi et al., 2001, 2005; Lohar et al., 2001; Sandal et al., 2002, 2012; Sato et al., 2008). These resources have facilitated molecular cloning of central genetic components required for endosymbiosis with rhizobia and arbuscular mycorrhiza (Schauser et al., 1999; Stracke et al., 2002; Radutoiu et al., 2003; Imaizumi-Anraku et al., 2005; Yano et al., 2008), positioning *L. japonicus* as a major model system for legume research. In a broader context, the *L. japonicus* resources were also exploited for comparative genomic approaches in a number of crop legumes, including pea, bean, and lupin, and for gene cloning in pea (Choi et al., 2004; Stracke et al., 2004; Fredslund et al., 2005; Hougaard et al., 2008; Li et al., 2010; McConnell et al., 2010; Nelson et al., 2010; Humphry et al., 2011; Krusell et al., 2011; Cruz-Izquierdo et al., 2012; Shirasawa et al., 2014).

Genetic analysis of natural variation within *L. japonicus* has now also been initiated. First, biparental populations have been used for QTL mapping of agronomic traits, host specificity, and nitrogen fixation efficiency using the three central experimental genotypes MG-20, Gifu and *L. burtii* (Gondo et al., 2007; Sandal et al., 2012; Tominaga et al., 2012), and diversity information has been exploited for evaluation the impact of Nod factor perception on rhizobium host range (Radutoiu et al., 2007; Bek et al., 2010). Second, a collection of ~200 *L. japonicus* accessions is managed and distributed by the Japanese National Bioresource Program (Hashiguchi et al., 2012). Phenotypic characterization has been carried out for some of these accessions (Kai et al., 2010) and so far 130 of them have been re-sequenced to facilitate GWAS analysis (Sato and Andersen, unpublished). There is a strong interest in the community for taking advantage of these new resources, and the accessions have been phenotyped for flowering time, trichome phenotype, nodulation capacity, salt tolerance, nematode resistance and root growth traits (Poch et al., 2007; Kubo et al., 2009; Gossmann et al., 2012; Wakabayashi et al., 2014; and unpublished results).

To take full advantage of GWAS-based genetic analysis and bring it to fruition in terms of functional characterization of genes, additional resources are needed. Expression data, high quality annotation and homology information is available for *L. japonicus*, facilitating quick selection of the most promising candidate genes for further investigation (Gonzales et al., 2005; Høgslund et al., 2009; Li et al., 2012; Verdier et al., 2013). Likewise, easy access to insertion mutants is critical to validate candidate gene involvement in a phenotype. Here, *L. japonicus* offers access to a unique resource in the non-transgenic LORE1 collection, which currently holds 40,000 annotated lines, with an additional 60,000 lines already characterized and soon to be released, bringing the total of annotated insertions to more than 500,000 (Urbanski et al., 2012; Andersen, unpublished). Together with the TILLING population this offers a comprehensive platform for mutant analysis (Perry et al., 2003).

The sum of these resources makes *L. japonicus* attractive as a model species, and the availability of non-transgenic insertion mutants offers the unique possibility of phenotyping *L. japonicus* and other legume diversity panels under similar conditions to estimate the degree of conservation of the genetic networks governing phenotypic responses across legumes and facilitating functional follow-up by characterization of candidate LORE1 mutant phenotypes under field conditions.

## Soybean benefits from comparison with its wild progenitors

Soybean is the most important legume crop and one of the largest global sources of vegetable oil and protein for people and livestock (Graham and Vance, 2003). Not surprisingly, the genome resource for soybean first published in 2010 is among the best for plants (Schmutz et al., 2010). For the initial assembly, more than 950 Mbp of the overall 1115 Mbp genome were completed through 8X Sanger shotgun sequencing and 98% could be anchored to specific chromosomal positions with few gaps. With this outstanding resource as a starting point, genome re-sequencing, genome-wide scans for selection, and GWAS have been fruitful in *Glycine* sp.

As a community-wide resource for genotyping a “SoySNP50K iSelect BeadChip” (Song et al., 2013) was designed from six cultivated and two wild soybean, aligned to the soybean genome reference. A total of 47,337 SNP calls were identified when tested against 96 landraces, 96 elite cultivars, and 96 wild accessions. This chip is used in many recent soybean GWAS publications.

Protein and oil content in the soybean seed are of special interest. In a GWAS analysis of 298 genotypes, Hwang et al. (2014) uncovered 40 associated SNPs in 17 different regions. One region was a long-sought protein/oil QTL on linkage group I (chromosome 20). This gene has been the target of QTL mapping research for many years, but earlier (biparental) studies struggled to localize the target gene to a region smaller than 8 Mbp (Bolon et al., 2011). In a second protein/oil GWAS study, Vaughn et al. (2014) discovered a more diagnostic and presumably more tightly linked locus as well as additional novel seed composition loci.

Further GWAS studies focused on abiotic and biotic stress. Concerning water deficit tolerance, Dhanapal et al. (2015) characterized carbon isotope ratios for 373 genotypes across four environments and 2 years, and described SNP tagging of 21 loci. Tolerance to low phosphorous is another important trait and GWAS enabled Zhang et al. (2014) to identify six regions associated with *P* efficiency. Closer examination of a region on chromosome 8 across 192 soybean accessions revealed a candidate, GmACP1 (an acid phosphatase). Favorable alleles and haplotypes of GmACP1 are associated with higher enzyme activity. Finally, resistance to the fungal pathogen *Fusarium virguliforme*, the cause of sudden death syndrome (SDS), was analyzed through GWAS in two association panels of elite cultivars by Wen et al. (2014). A total of 20 loci underlying SDS resistance were discovered, seven in regions previously described and 13 novel ones. One of these loci overlapped a previously cloned SDS resistance gene, *Rfs2*.

The availability of genome resources has enabled whole genome re-sequencing, focusing on soybean domestication from wild soybean (*G. soja*). For example, *G. soja* has been found to differ from the reference genome by only 0.31% (Kim et al., 2010). Re-sequencing 17 wild and 14 cultivated soybean genomes revealed unusually high levels of linkage disequilibrium (LD) with LD blocks greater than 1 Mb in cultivated soybean (Lam et al., 2010). Based on Fst scans across the genome, putative domestication regions (areas of high Fst) could be identified. Detailed mapping pinpointed smaller regions associated with known domestication traits such as twinning and stem elongation. By re-sequencing 55 accessions, Li et al. (2013) show that selection during early domestication led to more pronounced reduction in genetic diversity than the move from landraces to elite cultivars. Clusters of selection hotspots were observed involving 4.38% of total annotated genes. Finally, Li et al. (2014) initiated the first step toward a soybean pan-genome, generating *de novo* genome sequences independent of the soybean (*G. max* Williams-82) reference. This is important for characterizing structural variation, including copy number variation and for identifying core vs. dispensable portions of the soybean genome.

## Making the most of legumes – collaborations across species

To date, research efforts have focused on developing the resources, reference genome sequences and catalogs of natural diversity and germplasm, required to carry out association studies within a species. With the successful establishment of these prerequisites, the time has come to consider the possibilities offered by the resources, along with those emerging in multiple legume species. While GWAS is a powerful tool for genetic analysis and causal allele identification, it also comes with inherent challenges. Inaccuracies in geno- and phenotyping combined with population sampling and structure can all lead to false positive signals. However, these confounding factors are species-specific, as the legume resources have largely been developed independently for each species. As such, genotype-phenotype associations detected recurrently in at least two legume species would therefore be strongly mutually supportive.

Leveraging these potential gains in GWAS candidate gene confidence will require a new paradigm for GWAS phenotyping, where multi-site, multi-year phenotyping strategies are combined with multi-species trials. Approaches where multiple legume species are grown side-by-side for phenotyping at multiple geographical locations will not be relevant for all traits, but obvious traits suitable for analysis are abiotic and broad host-range biotic stresses where similar adaptive strategies could be in play across species. Such phenotyping strategies have the potential to break new ground in the understanding of environmental adaptation, but organization of international consortia that could successfully implement these approaches would require a high level of (re-)organization, for researchers, breeders, and funding bodies.

### Conflict of interest statement

The authors declare that the research was conducted in the absence of any commercial or financial relationships that could be construed as a potential conflict of interest.

## Footnotes

^1^http://faostat3.fao.org/browse/Q/QC/E
